# Posterosuperior shoulder dislocation due to the rupture of deltoid posterior fibers: a case report

**DOI:** 10.1186/s12891-019-2727-5

**Published:** 2019-07-27

**Authors:** Ryogo Furuhata, Yasuhiro Kiyota, Taiki Ikeda, Masaaki Takahashi, Hideo Morioka, Hiroshi Arino

**Affiliations:** Department of Orthopaedic Surgery, National Hospital Organization Tokyo Medical Center, 2-5-1, Higashigaoka, Meguro-ku, Tokyo, 152-8902 Japan

**Keywords:** Superior shoulder dislocation, Conservative treatment, Deltoid rupture, Rotator cuff tear

## Abstract

**Background:**

Superior shoulder dislocation is a rare type of shoulder dislocation. Its occurrence is thought to be associated with rupture of the deltoid; however, few reports are available on the mechanism of onset and the treatment of a superior shoulder dislocation. Here we describe a case of dislocation in the direction of the posterior acromion, referred to as posterosuperior shoulder dislocation, caused by the traumatic rupture of deltoid posterior fibers.

**Case presentation:**

An 83-year-old woman who fell on her right elbow in the hospital presented to our department with complaints of right shoulder pain. She had been undergoing conservative treatment for a massive rotator cuff tear and a rupture of the long head of biceps tendon 5 years previously. X-ray radiography images at the time of the trauma revealed that the right humeral head was dislocated upwards and in the direction of the posterior acromion. Magnetic resonance imaging (MRI) revealed newly ruptured deltoid posterior fibers, in addition to the massive rotator cuff tear. Closed reduction was performed and the shoulder joint was held in external rotation at 30 degrees for 6 weeks. However, re-dislocation was observed at an early stage after the removal of the orthosis and marked instability remained.

**Conclusions:**

This is the first case of posterosuperior shoulder dislocation. It suggests that rupture of the deltoid posterior fibers contributes to the onset of posterosuperior shoulder dislocation in patients with a massive rotator cuff tear. Moreover, in superior shoulder dislocation, conservative treatment may result in continuing instability which requires surgical treatment.

## Background

Anterior or posterior shoulder dislocations are the most commonly seen of all shoulder dislocations, and superior shoulder dislocation is extremely rare [1]. Previous reports on superior shoulder dislocation to date include anterosuperior dislocation, in which the humeral head dislocates in the direction of the anterior acromion [2–5]; superolateral shoulder dislocation, in which the humeral head dislocates in the direction of the lateral acromion [6, 7]; and locked superior shoulder dislocation, in which the humeral head dislocates and is locked in the space between the superior margin of the glenoid and acromion [8]. While it has been reported that the occurrence of superior shoulder dislocation is associated with deltoid rupture [4, 5, 7] or deltoid contracture [3], due to its rarity, little is known about its mechanism of onset and risk factors. Additionally, few reports are available on its treatment and prognosis.

Here, we report a case of posterosuperior shoulder dislocation caused by a combination of rupture of deltoid posterior fibers and a massive rotator cuff tear. This is the first case of dislocation in the direction of the posterior acromion. Since the patient refused surgery, conservative treatment was provided; however, instability remained. This case suggests that surgical treatment is required in the case of a superior shoulder dislocation accompanied by a deltoid rupture.

## Case presentation

**Fig. 1:**
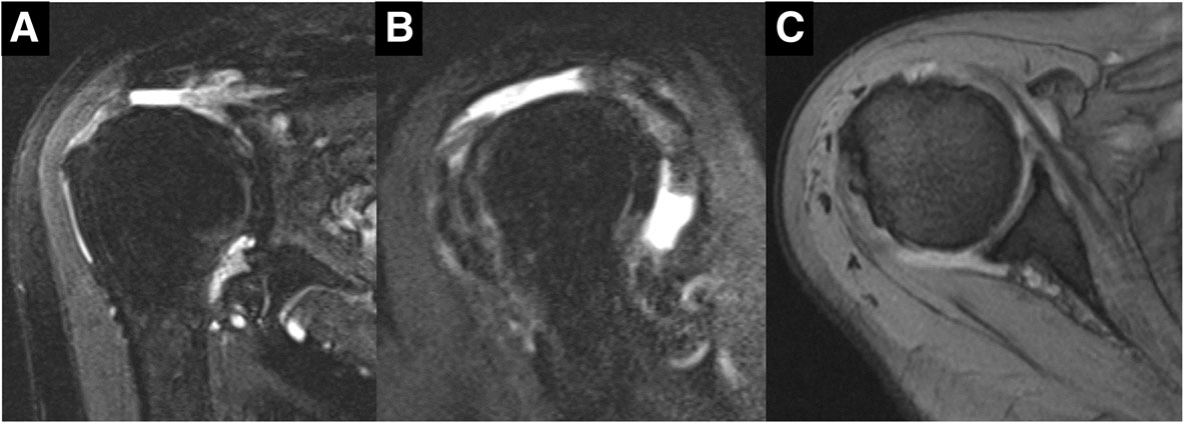
MRI images of the right shoulder 2 years before the trauma. Fat-suppressed T2-weighted oblique axial (**a**) and oblique sagittal (**b**) images, and T2-weighted oblique coronal image (**c**) revealing a complete tear and retraction of the supraspinatus tendon and a rupture of the long head of the biceps tendon. MRI, magnetic resonance imaging

**Fig. 2:**
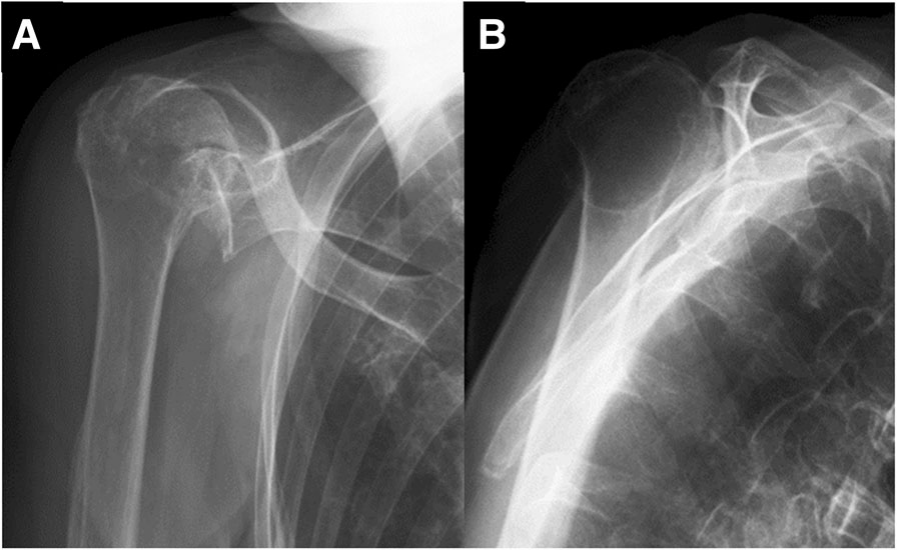
X-ray radiography images demonstrating the right humeral head dislocated upward in a direction posterior to the acromion (**a**, **b**)

**Fig. 3:**
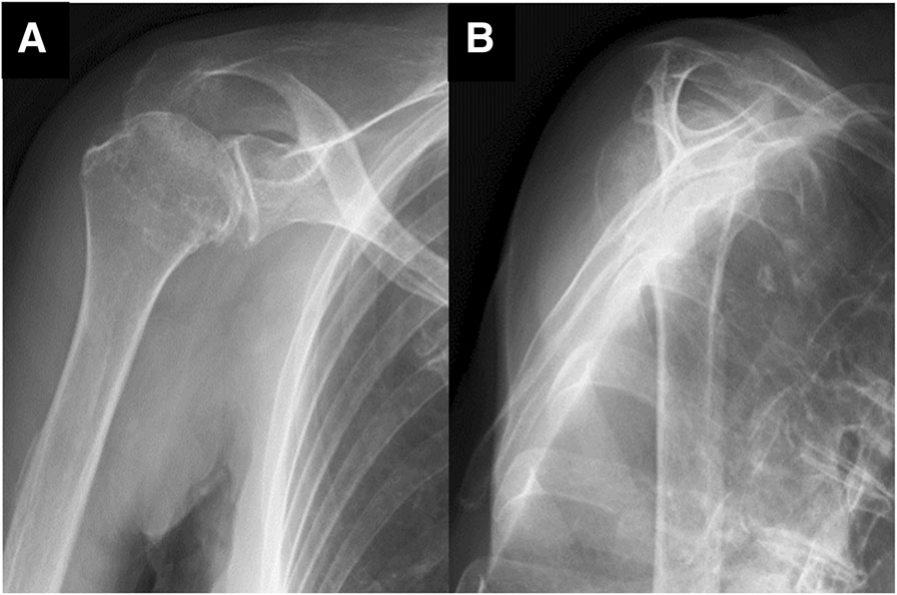
X-ray radiography images after reduction revealing that the dislocation was reduced (**a**, **b**)

**Fig. 4:**
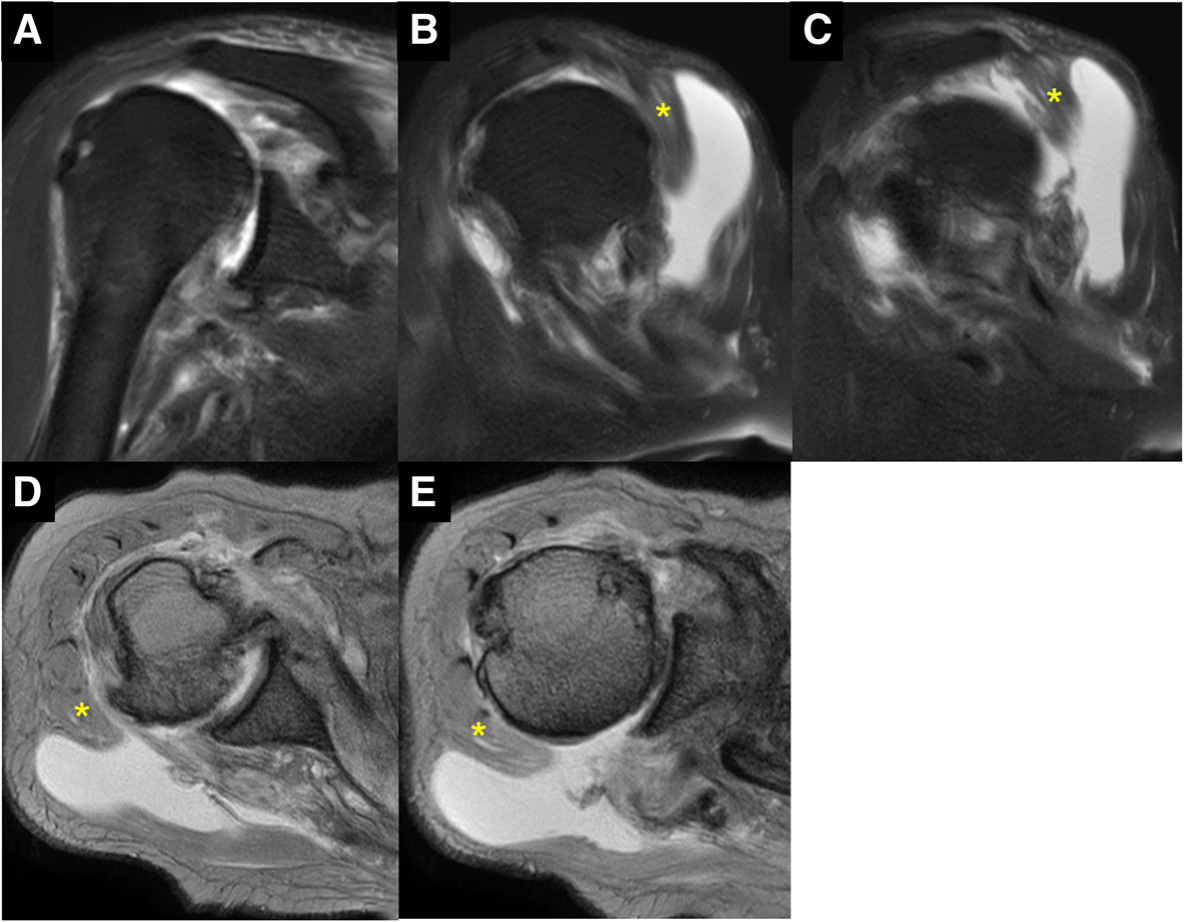
MRI images of the right shoulder 1 week after the closed reduction. Fat-suppressed T2-weighted oblique axial (**a**) and oblique sagittal (**b**, **c**) images, and T2-weighted oblique coronal images (**d**, **e**) revealing the complete tear of supraspinatus tendon and infraspinatus tendon, rupture of deltoid posterior fibers (asterisk), and fluid retention in the posterosuperior shoulder. MRI, magnetic resonance imaging

**Fig. 5:**
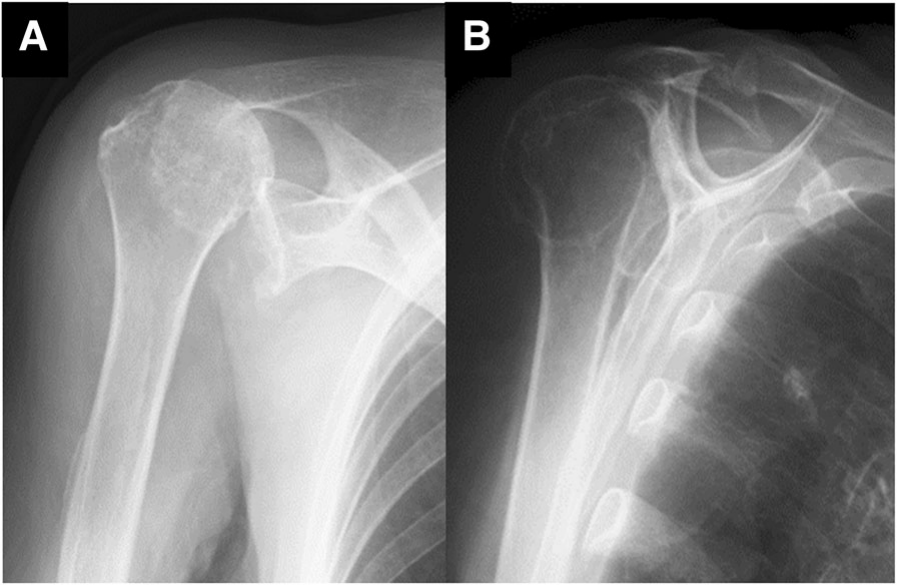
X-ray radiography images soon after the removal of orthosis revealing re-dislocation (**a**, **b**)

An 83-year-old woman who fell on her right elbow in the hospital, presented to our department with complaints of right shoulder pain. The patient had been undergoing conservative treatment for a massive rotator cuff tear and rupture of the long head of the biceps tendon 5 years previously. Magnetic resonance imaging (MRI) taken 2 years prior to the trauma showed a complete tear and retraction of the supraspinatus tendon and a rupture of the long head of the biceps tendon (Fig. 1). In addition, the patient had been diagnosed by a nephrologist as having anti-neutrophil cytoplasmic antibody (ANCA)-associated microscopic polyangiitis 2 months prior to the trauma, and was treated with oral prednisolone for 2 months. Physical examination findings at the time of the trauma demonstrated difficulty in moving the right shoulder due to pain. The humeral head was palpable posterior to the acromion. There were no findings suggestive of nerve or vascular injury. X-ray radiography images revealed that the right humeral head was dislocated upwards and posterior to the acromion (Fig. 2). A closed reduction was performed by traction and adduction of the upper extremity, and by pushing the coracoid process. Reduction was easily performed, and X-ray radiography images after the reduction revealed that the dislocation had been reduced (Fig. 3). An MRI 1 week after the shoulder dislocation showed the newly ruptured deltoid posterior fibers and infraspinatus tendon, in addition to a supraspinatus tear and a biceps tendon rupture, and fluid retention in the posterosuperior shoulder (Fig. 4). Following the closed reduction, the shoulder joint was held in external rotation at 30 degrees with an external rotation orthosis for 6 weeks. However, re-dislocation was observed at an early stage following the removal of the orthosis (Fig. 5). While reduction was performed as required, dislocation frequently recurs due to movement such as changing clothes. Although surgical treatment was recommended, the patient and her family refused it; therefore, conservative treatment was continued. At 3 months following the trauma, while the right shoulder pain had subsided, the range of motion was limited to forward elevation of 20 degrees, and the patient was having difficulty eating with her right hand.

## Discussion and conclusion

The present case highlights two clinical issues. First, our case suggests that posterior deltoid rupture contributes to the onset of posterosuperior shoulder dislocation in patients with massive rotator cuff tears. Previous reports state that superior shoulder dislocation occurs together with deltoid rupture and massive rotator cuff tears. It has been reported that anterosuperior shoulder dislocation, in which dislocation occurs in the direction anterior to the acromion, is caused by a combination of rotator cuff insufficiency including the supraspinatus and subscapularis tendons, a loss of the coracoacromial arch, and a rupture of deltoid anterior fibers [4, 5]. In addition, it has been reported that superolateral dislocation, in which dislocation occurs in the direction of the lateral side of the acromion, is caused by a longitudinal split tear of the anterolateral deltoid muscle and a massive rotator cuff tear [7]. However, dislocation in the direction of the posterior acromion, namely posterosuperior dislocation, was observed for the first time in the present case. Here, the imaging findings suggested that a new traumatic rupture of the posterior deltoid fibers, in addition to the underlying chronic massive rotator cuff tear, may have caused the posterosuperior shoulder dislocation. Since there is no osseous-tissue barrier, but only soft-tissue barriers such as the rotator cuff, articular capsule, and deltoid posterior fiber for the posterosuperior direction of the shoulder joint, when a patient with a chronic massive rotator cuff tear additionally suffers a rupture of the deltoid posterior fibers, this barrier mechanism appears to break down easily, leading to the posterosuperior dislocation. A deltoid rupture usually occurs as a complication of rotator cuff surgeries, and there have been few reports of spontaneous deltoid ruptures. It has been reported that repeated intra-tendon injections of steroids may play a role in the development of spontaneous deltoid ruptures [9, 10]. Although the present patient did not have a past history of steroid injections into the shoulder, the oral administration of high-dose steroids had commenced for ANCA-associated vasculitis 2 months earlier, and this may have increased the risk of deltoid rupture. Additionally, it has been speculated that chronic massive rotator cuff tears may affect the occurrence of a deltoid rupture as a subsequent mechanism [10]. A massive rotator cuff tear is thought to cause the humeral head, in particular the greater tubercle, to shift upward, which causes the greater tubercle to impinge on the undersurface of the deltoid muscle during shoulder movement. This friction causes degeneration of deltoid muscle fibers and increases the risk of deltoid rupture [10]. In this case, a massive tear of the supraspinatus tendon may cause impingement of the greater tubercle on the undersurface of the deltoid muscle, leading to the deltoid rupturing with subsequent posterosuperior dislocation.

Second, in cases of superior shoulder dislocation accompanying a deltoid rupture, conservative treatments may result in a poor outcome which would require surgical treatment. Previous reports on the treatment and outcome of traumatic superior shoulder dislocation are summarized in Table 1. In the previous reports on conservative treatment for superior dislocation, early reduction and immobilization were attempted; however, it has been reported that reduction itself was difficult [2] and that re-dislocation was observed following reduction and immobilization for 4 weeks [8]. In the present case, surgical treatment would have been optimal because the instability remained despite 6 weeks of external rotation fixation. These reports suggest that it is difficult to achieve shoulder joint stability in cases of superior shoulder dislocation with conservative treatment alone. Surgical options for superior shoulder dislocations include rotator cuff repair or reverse shoulder arthroplasty, in addition to deltoid repair. While there is a report stating that open reduction and rotator cuff repair achieved reduction in a young patient with superolateral dislocation [7], another report states that early re-dislocation was observed in an elderly patient with Parkinson’s disease suffering from anterosuperior dislocation, and a rotator cuff repair was performed [5]. Therefore, the outcome of rotator cuff repair for superior shoulder dislocation remains controversial and further research and investigation is needed. Reverse shoulder arthroplasty has been used for massive rotator cuff tears; however, deltoid dysfunction is considered to be a contraindication and precludes its use. On the other hand, there is a report that shows a satisfactory result 2 years postoperatively after reverse shoulder arthroplasty in a patient suffering from a deltoid rupture and rotator cuff tear arthropathy in the absence of superior shoulder dislocation [11]. In addition, it has also been reported that reverse shoulder arthroplasty associated with repair of the deltoid was performed in 18 elderly patients with massive irreparable rotator cuff tears and associated rupture of the anterior and middle deltoid muscle, and that satisfactory clinical outcomes were achieved in the medium term, except for the patients with preoperative chronic axillary nerve neuropathy [12]. These reports suggest that reverse shoulder arthroplasty can be a surgical option for patients with deltoid ruptures and massive rotator cuff tears. In the present case, surgical treatment was not selected at the patient’s request, and marked instability remained. Reverse shoulder arthroplasty may be one of the surgical options to restore shoulder stability and function.

**Table 1:** Summary of previous reports on the treatment and outcome of traumatic superior shoulder dislocation

Author	Age / Sex	Type of Dislocation	Closed Reduction	Treatment	Outcome
De Laat EA et al. (1997)	88 / Female	Anterosuperior	Irreducible	N.D.	N.D.
Matuzaki T et al. (2008)	83 / Female	Anterosuperior	Reducible	Rotator Cuff Repair	Re-dislocation + (10 weeks after injury)
Wyatt AR et al. (2015)	53 / Male	Superolateral	Irreducible	Rotator Cuff Repair	N.D.
Plachel F et al. (2018)	61 / Female	Locked Superior	Reducible	Immobilization (4 weeks)	Re-dislocation + (8 weeks after injury)
Our Case (2018)	83 / Female	Posterosuperior	Reducible	Immobilization (6 weeks)	Re-dislocation + (6 weeks after injury)

N.D., Not described

In conclusion, the present case provides important information on the functional significance of the posterior deltoid muscle in the regulation of posterosuperior instability of the shoulder joint. Surgical treatment would be recommended in the case of posterosuperior shoulder dislocation accompanying deltoid rupture, as in this case.

## Data Availability

Data that support the findings of this study are available from the corresponding author on reasonable request.

## Abbreviations

ANCA: Anti-neutrophil cytoplasmic antibody
MRI: Magnetic resonance imaging
